
JOURNAL INFORMATION
==============================
NLM Title Abbreviation: Pain Res Manag
Journal ID: Pain Res Manag
NLM Journal ID: 9612504
Journal ID: PRM

Pain Research & Management

ISSN: 1203-6765
EISSN: 1918-1523
Publisher: Wiley

ARTICLE INFORMATION
==============================
PMCID: PMC5499488
PMID: 19532854
DOI: 10.1155/2009/698624
Article ID: 698624
Article version: 1
Subjects: Letter to the Editor

Re: KD Craig. Knowledge Translation and the Science of Pain. Pain Res Manage 2008;13(6):464

Daoust Robert
Algosphere Montreal Quebec Canada
Print publication date: 2009 Mar-Apr
Volume: 14
Issue: 2
First page: 173
Last page: 173
Copyright: Copyright © 2009 Hindawi Publishing Corporation.
Copyright year: 2009
License: This is an open access article distributed under the Creative Commons Attribution License, which permits unrestricted use, distribution, and reproduction in any medium, provided the original work is properly cited.
License URL: https://creativecommons.org/licenses/by/4.0/

Comment on: Knowledge translation and the science of pain 13 6 Nov-Dec 2008 464 464 Pain Research & Management : The Journal of the Canadian Pain Society 10.1155/2008/151970 PMC2799314 19225602

==============================
### LETTER TO THE EDITOR

**Re: Craig KD. Knowledge translation and the science of pain. Pain Res Manage 2008;13:464.**

Bonjour docteur Craig,
Your editorial about knowledge translation prompts me to send you this message.

I believe the problem of pain science knowledge translation has a political dimension that should be confronted head-on. It is a problem of resource distribution that could be compared, for example, with the one that prevails in nutrition science, in which solutions to hunger are well known but can only be implemented through politically adequate resource distribution.

What modern politics are still lacking, in my view, is an approach to suffering (algonomy; www.algosphere.org/indexen.htm) that could inform social-economic management. There is a need for a science of suffering (algoscience; www.algosphere.org/intro/index.html), and the fields of psychology and pain science should be more aware of that need. Your editorial uses the words 'suffer' and 'suffering', it raises the question of how pain is conceptualized and it states that there is a great need for integrative, even speculative, reviews and theoretical analyses. I would like us to go further – to clarify the link between pain and suffering, to recognize that because pain is an unpleasant experience, it is therefore a kind of suffering, to perform not only reviews and analyses but also to create the new science of suffering that must be created.

If you ever hear of someone who might be interested, able and available for that kind of creation work, please let me know!

*Robert Daoust*
*Algosphere*
*Montreal, Quebec*
